# Validation of the Dutch version of the quick mild cognitive impairment screen (Q*mci*-D)

**DOI:** 10.1186/s12877-015-0113-1

**Published:** 2015-10-02

**Authors:** Steven Bunt, Rónán O’Caoimh, Wim P. Krijnen, D. William Molloy, Geert Pieter Goodijk, Cees P. van der Schans, Hans J S M Hobbelen

**Affiliations:** 1 Research group Healthy Ageing, Allied Health Care and Nursing, Hanze University of Applied Sciences, Eyssoniusplein 18, P.O. Box 30030, 9700 RM Groningen, The Netherlands; 2 Centre for Gerontology and Rehabilitation, University College Cork, St Finbarrs Hospital, Douglas Road, Cork City, Ireland; 3 Health Research Board, Clinical Research Facility Galway, National University of Ireland, Galway, Ireland; 4 Department of Rehabilitation, University Medical Center Groningen, University of Groningen, Groningen, The Netherlands

**Keywords:** Validity, Mild cognitive impairment, Dementia, Quick mild cognitive impairment screen, Screening

## Abstract

**Background:**

Differentiating mild cognitive impairment (MCI) from dementia is important, as treatment options differ. There are few short (<5 min) but accurate screening tools that discriminate between MCI, normal cognition (NC) and dementia, in the Dutch language. The Quick Mild Cognitive Impairment (Q*mci*) screen is sensitive and specific in differentiating MCI from NC and mild dementia. Given this, we adapted the Q*mci* for use in Dutch-language countries and validated the Dutch version, the Q*mci*-D, against the Dutch translation of the Standardised Mini-Mental State Examination (SMMSE-D).

**Method:**

The Q*mci* was translated into Dutch with a combined qualitative and quantitative approach. In all, 90 participants were recruited from a hospital geriatric clinic (25 with dementia, 30 with MCI, 35 with NC). The Q*mci*-D and SMMSE-D were administered sequentially but randomly by the same trained rater, blind to the diagnosis.

**Results:**

The Q*mci*-D was more sensitive than the SMMSE-D in discriminating MCI from dementia, with a significant difference in the area under the curve (AUC), 0.73 compared to 0.60 (*p* = 0.024), respectively, and in discriminating dementia from NC, with an AUC of 0.95 compared to 0.89 (*p* = 0.006). Both screening instruments discriminated MCI from NC with an AUC of 0.86 (Q*mci*-D) and 0.84 (SMMSE-D).

**Conclusion:**

The Q*mci*-D shows similar,(good) accuracy as the SMMSE-D in separating NC from MCI; greater,(albeit fair), accuracy differentiating MCI from dementia, and significantly greater accuracy in separating dementia from NC. Given its brevity and ease of administration, the Q*mci*-D seems a useful cognitive screen in a Dutch population. Further study with a suitably powered sample against more sensitive screens is now required.

## Background

The prevalence of mild cognitive impairment (MCI) [1] and dementia [2] is increasing worldwide. Differentiating MCI from dementia is important, because treatment options differ. In particular, pharmaceutical therapy, indicated for treatment of dementia, is inappropriate and potentially even harmful, in those with MCI [3]. Differentiating MCI from normal cognition (NC) is also important, because people with MCI are at increased risk of developing dementia, compared to aged-matched individuals in the population [4], and early identification may allow prompt intervention [5, 6]. Few short (administration time of approximately 5 min) cognitive screening instruments are useful in discriminating between MCI and NC or dementia. Most are limited by their sensitivity and specificity [7]. Likewise, few are available in the Dutch language. One of the most widely used tools is the Mini-Mental State Examination (MMSE) [8]. The MMSE was standardized to improve reliability, producing the Standardised Mini-Mental State Examination (SMMSE) [9, 10], which is available in a wide variety of languages including Dutch [11]. However, there is limited evidence that either the MMSE or SMMSE are sufficiently accurate in identifying MCI [12], particularly in those with high educational attainment [13].

To address these challenges, the Quick Mild Cognitive Impairment (Q*mci*) screen was developed. Based upon the ABCS 135 [14], it was modified to increase its sensitivity to differentiate NC from MCI, by the addition of logical memory. The Q*mci* is more sensitive in differentiating MCI from NC than the SMMSE and ABCS 135 [15]. The Q*mci* has six subtests, orientation, registration, clock drawing, delayed recall, verbal fluency and logical memory. It is scored out of 100 points, can be administered and scored in less than 5 min and has excellent test-retest reliability [16]. The Q*mci* correlates highly with the Standardised ADAS-cog, Clinical Dementia Rating scale and the Lawton-Brody activities of daily living scale [17].

The goal of the present study was to adapt the Q*mci* for use in Dutch-language countries, to validate the Dutch version of the Q*mci* (Q*mci*-D) and to compare its sensitivity and specificity in differentiating MCI from NC and dementia to the most widely used short cognitive screen in the Netherlands, the Dutch version of the SMMSE (SMMSE-D).

## Methods

### Translation

The translation of the Q*mci* was performed with a combined qualitative and quantitative approach [18]. The original version of the Q*mci* was translated to Dutch by a health professional with a good understanding of English, whose primary language is Dutch. This Dutch version was reviewed by an expert panel of Dutch health professionals and researchers and a completed version of the Q*mci*-D was generated. A professional, native English language-speaking translator, without knowledge of the concepts behind the screening tool, performed the back-translation. The back-translation was then reviewed by the original developers of the Q*mci* screen, who approved the final version. The Q*mci*-D was pre-tested on participants with normal cognition before it was used in this study.

### Participants

Consecutive patients were recruited during a four month-period, between November 2013 and March 2014, from a geriatric outpatient department in a regional hospital, in the North of the Netherlands, where they were referred for the assessment of cognitive problems. Normal controls were recruited by convenience sampling among healthy participants without cognitive problems, who were accompanying the patients. A diagnosis of dementia (Alzheimer’s disease, vascular or mixed dementia subtypes) was made using DSM-IV [19] and NINCDS-ADRDA [20] criteria. A diagnosis of amnestic type MCI was made in patients with objective memory loss, greater than expected with ageing but without loss of social or occupational function, according to the National Institute on Aging- Alzheimer’s Association workgroups diagnostic guidelines for Alzheimer’s disease [21]. Participants were excluded if they were younger than 55, if they had active depression (Geriatric Depression Scale >5), if they weren’t able to communicate in Dutch or if they were diagnosed with other MCI or dementia subtypes, including frontotemporal dementia, Parkinson’s disease or Lewy Body dementia. Those with frontotemporal, Parkinson’s disease and Lewy body dementia were excluded as they present infrequently [22] and typically present with exaggerated functional deficits and different MCI syndromes [23–25].

The MCI and dementia groups underwent the same comprehensive review at baseline including neuropsychological assessment and Magnetic Resonance Imaging or Computerized Tomogram. The purpose and procedure of the research were explained in advance and all participants signed an informed consent before participation in the study. The Medical Ethical Committee of the University Medical Center Groningen evaluated the study and judged that it did not need ethical approval under Dutch law.

A power calculation was performed *a priori,* to establish the sample size, using the WINPEPI software programme PAIRSetc [26, 27]. Based upon the original validation results of the Q*mci* compared to the SMMSE [15], it was expected that the accuracy, as indicated by the area under the curve (AUC) of receiver operating characteristic (ROC) curves, of the Q*mci* would be 85 % compared to approximately 65 % for the SMMSE, to differentiate MCI from NC. To detect a 20 % (medium to large effect size) difference in sensitivity and specificity, between the two tests, at a significance level of 0.05 and power of 80 %, 76-paired observations were required. The sample size needed to distinguish MCI from dementia was not estimated as a significant difference between the Q*mci* and the SMMSE would not be expected [15].

### Data collection

Demographic data (age and gender) were collected during each visit to the geriatric department. Patients were classified by a consultant geriatrician and divided into three groups (NC, MCI or dementia). A trained rater administered both the Q*mci*-D (score range 0–100, impaired to normal) and SMMSE-D (score range 0–30, impaired to normal) on the same day, in a counterbalanced fashion, blind to the final diagnosis.

### Statistical analysis

Data were analyzed using SPSS version 20.0 and the statistical programming language R. The Shapiro-Wilk test was used to test for normality. Differences in Q*mci*-D and SMMSE-D scores, between groups, were tested by one-way analysis of variance (ANOVA). Analysis of covariance (ANCOVA) was used to test differences between participant groups, controlling for participant characteristics such as age. Post hoc pair-wise comparisons were performed using the Tukey’s honest significant difference (HSD) test. A p-value < 0.05 was regarded as significant. Bootstrapped ROC curves were generated [28] to analyze the discriminatory characteristics of the Q*mci-*D and SMMSE-D [29]. Differences between AUCs were calculated using the DeLong approach [30].

## Results

In total, 90 participants, 41 males (46 %) and 49 females (54 %), were included in this study. In this sample, 35 (39 %) had NC, 30 (33 %) had MCI and 25 (28 %) were diagnosed with dementia. The overall mean age of the sample was 72.9, standard deviation (SD) of 9.1 years. The NC group (mean age 68.7) was younger than the MCI (mean age 79.1) and dementia (mean age 79.2) groups (*p* < 0.001). The NC group had a mean Q*mci*-D score of 64 (SD 10.5) and a mean SMMSE-D score of 28 (SD 1.8). The mean Q*mci*-D score for the MCI group was 46 (SD 11.8) compared with 24 (SD 2.9) for the SMMSE-D, while the dementia group scored 34 (SD 15.8) for the Q*mci*-D and 22 (SD 4.4) for the SMMSE-D. These scores and demographic data are summarized in Table 1.

**Table 1:** Characteristics of patients with mild cognitive impairment (MCI), dementia, and participants with normal cognition (NC) including their Quick Mild Cognitive Impairment (Q*mci*-D) screen and Standardised Mini-Mental State Examination (SMMSE-D) scores

Group	Dementia	MCI	Normal cognition
Number of participants	25	30	35
Age			
Mean (SD)	79.2 (5.7)	79.1 (5.9)	68.7 (9.0)
Median (IQR)	80 (84–76 = 8)	80 (83–74 = 9)	68 (77–61 = 16)
Gender			
(% female)	48 %	56 %	57 %
Q*mci*-D (range 0–100)			
Mean Score (SD)	34 (15.8)	46 (11.8)	64 (10.5)
Median Score (IQR)	35.5 (48–21 = 17)	46.8 (54–38 = 16)	64.5 (72–55 = 17)
SMMSE-D (range 0–30)			
Mean Score (SD)	22 (4.4)	24 (2.9)	28 (1.8)
Median Score (IQR)	23 (26–18 = 8)	23.5 (26–22 = 4)	28 (29–27 = 2)

*SD* Standard Deviation, *IQR* inter-quartile range, *IQR* Q1-Q3, *Q1* 1st Quartile, *Q3* 3rd quartile

**Fig. 1:**
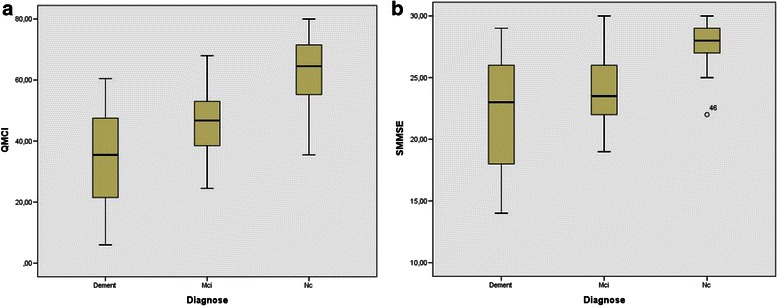
Boxplots representing scores on the (**a**) Q*mci*-D (score range 0–100) and (**b**) SMMSE-D (score range 0–30) in dementia, MCI and normal cognition groups

One-way ANCOVA, used to test for differences in SMMSE-D scores between the three groups (NC, MCI and dementia), showed that the mean scores differed significantly across the three groups (*F* = 20.55, df = 3,86, *p* < 0.001). Post hoc pair-wise comparisons using the Tukey’s HSD test showed significant mean differences between groups (*p* < 0.05). The mean scores for the Q*mci*-D also differed significantly between the three groups; (*F* = 33.24, df = 3,86, *p* < 0.001). The differences between groups are presented as boxplots in Fig. 1. ANOVA post-hoc testing for multiple comparisons, showed a significant difference in mean scores between the dementia and NC groups, for both tests (see Table 2).

**Table 2:** ANOVA Post-Hoc tests: multiple comparisons between NC, MCI and Dementia groups

Dependent Variable	Group	Group	Mean Difference	Std. Error	*P*-value	95 % Confidence Interval	
						Lower Bound	Upper Bound
Q*mci*-D	MCI	Dementia	12.56	3.40	0.001	4.25	20.87
	NC	MCI	17.19	3.13	<0.001	9.55	24.82
	NC	Dementia	29.75	3.29	<0.001	21.71	37.78
SMMSE-D	MCI	Dementia	1.79	0.83	0.102	−0.24	3.83
	NC	MCI	3.70*	0.77	<0.001	1.84	5.58
	NC	Dementia	5.50*	0.81	<0.001	3.54	7.47

*SMMSE-D* Dutch version of the Standardised Mini Mental State Examination, Q*mci*-*D* Dutch version of the Quick Mild Cognitive Impairment Screen, *MCI* Mild Cognitive Impairment, *NC* Normal Cognition

**Fig. 2:**
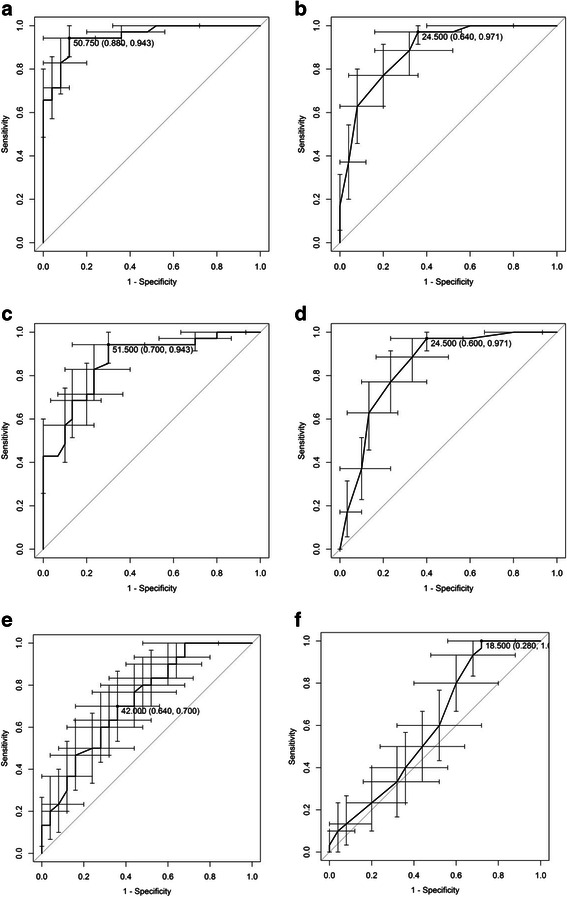
Bootstrapped ROC curves with 95 % confidence intervals demonstrating sensitivities and specificities of (**a**) Q*mci*-D and (**b**) SMMSE-D in differentiating dementia from normal cognition, the (**c**) Q*mci*-D and (**d**) SMMSE-D in differentiating MCI from normal cognition, and the (**e**) Q*mci*-D and (**f**) SMMSE-D in differentiating MCI from dementia

Comparisons of the AUC of ROC curves and the point estimates (cut-off scores), providing the optimal sensitivity and specificity, are presented in Fig. 2. These show that the Q*mci*-D was more accurate than the SMMSE-D in discriminating between dementia and NC, with an AUC of 0.95 (95 % CI 0.90–0.99), compared to 0.89 (95 % CI 0.80–0.96) for the SMMSE-D (see Fig. 2a, b). The difference was significant (*p* = 0.006). Both the Q*mci*-D and the SMMSE-D discriminated MCI from NC, with ANOVA post-hoc tests showing a significant mean difference between the MCI and NC groups (*p* < 0.001). The AUC of the Q*mci*-D, in discriminating MCI from NC, was marginally greater at 0.86 (95 % CI 0.77–0.95), compared to 0.84 (95 % CI 0.74–0.94) for the SMMSE-D (see Fig. 2c, d). This difference was non-significant (*p* = 0.335). At the point estimate the Q*mci*-D had a sensitivity of 70 % and specificity of 94 % compared to a sensitivity of only 60 % and a similar specificity of 94 % for the SMMSE-D. As for the discrimination of MCI from dementia, ANOVA post-hoc tests, showed a significant mean difference (*p* < 0.001) between these participants with the Q*mci*-D. The difference between scores for the MCI and dementia groups, for the SMMSE-D (*p* = 1.02) was not significant. The score test for homogeneity of variances across groups in ANCOVA indicated that homogeneity for the Q*mci* is rejected. Heteroscedasticity-corrected SEs and Tests after ANCOVA [31] indicated that the difference in Q*mci*-D mean scores after correction for age, between MCI and NC as well as between MCI and dementia, were significantly different from zero. The ability of the Q*mci*-D, to discriminate MCI from dementia, was significantly greater (*p* = 0.024) than the SMMSE-D, AUC of 0.73 (95 % CI 0.59–0.85) compared to 0.60 (95 % CI 0.45–0.75), respectively (See Fig. 2e, f). At the point estimate the Q*mci*-D had a modest sensitivity of 64 % to differentiate MCI from dementia, although this compared favorably to the SMMSE-D with a sensitivity of only 28 %.

When moderate and severe dementia cases were removed from analysis, the AUC of the Q*mci*-D and SMMSE-D for differentiating MCI from mild dementia cases alone was unchanged at 0.62 and 0.54, *p* = 0.03, respectively.

## Discussion

The goals of this study were to adapt the Q*mci* for use in Dutch-language countries and to explore its concurrent validity against the most commonly used short cognitive screen in Dutch, the SMMSE-D. The results show that the Q*mci*-D is more accurate than the SMMSE-D in differentiating dementia from NC. It had only fair accuracy (AUC 0.73) at differentiating MCI from dementia, although it was significantly more accurate than the SMMSE (AUC 0.60). In this study however, the SMMSE-D wasn’t able to discriminate MCI from dementia with a very poor sensitivity of 24 %, particularly when moderate to severe cases were excluded. Based upon this data it would appear that both instruments have limited ability to separate MCI from dementia. This is markedly different from the initial validation of the English language version of the Q*mci* against the SMMSE [15], which in a much larger sample of almost 1000 Canadians, suggested that both had excellent accuracy (AUC >0.90), although it showed no significant difference between the two instruments in their ability to distinguish MCI from dementia. There was also no significant difference between the tests’ ability to discriminate between MCI and NC in this sample, although the accuracy of both tests was good, suggesting that both were able to separate MCI from NC. This again differs from the initial validation, where the Q*mci* showed significantly greater accuracy over the SMMSE. This discrepancy may relate to the small sample size, suggesting that this study was underpowered to show superiority of one instrument over the other. This said, the goal of this study was not to show superiority of a Dutch language version of the Q*mci*, the Q*mci*-D, rather it was to show the concurrent validity of the translation against a widely used screening instrument.

The strength of this study is the robust analysis with bootstrapped ROC curves and 95 % confidence intervals, to identify the discriminatory characteristics of both screening tools. This method provides more accurate results than non-bootstrapped methods, especially when analyzing smaller sample sizes. The 95 % confidence intervals obtained from the bootstrap and the asymptotic approach [30], were in all cases virtually equal. This indicates that the intervals are valid.

The study has limitations. First, the diagnosis of MCI was based on clinical criteria, which may have increased the heterogeneity of this group and led to some bias. However, no single gold standard criterion for MCI exists and there is still a lack of uniformity in the clinical diagnosis of MCI between studies [32]. This said, in this study an objective history of cognitive decline over time was obtained from each patient’s collateral (family member or caregiver) and assessed by neuropsychological testing, independent of the results of the short cognitive screens, in keeping with the National Institute on Aging Alzheimer’s Association diagnostic guidelines [21]. Second, the NC group consisted of participants recruited by convenience sampling from healthy relatives or caregivers attending with patients. These participants were significantly younger than patients with MCI or dementia. This could have increased heterogeneity and created bias, explaining why there was no significant difference in the ability of both instruments to distinguish MCI from NC, unlike that seen in the initial validation of the Q*mci* [15]. Patients with MCI and dementia were, however, well matched for age and gender. ANCOVA testing and post-hoc analysis confirmed that differences in mean test score were not attributable to age. Furthermore, the educational status of patients was not recorded routinely, which may also have created some bias. Third, the sample size was small and did not exceed the desired 76-paired observations, calculated as the sample size to detect differences in accuracy between participants with MCI and NC for the screening tools. Fourth, the study excluded those with active depression and less prevalent dementia subtypes as described above. Active depression was excluded as these patients may have slower reaction times and processing speeds [33]. Frontotemporal, Parkinson’s disease and Lewy body dementia often present with exaggerated functional deficits potentially clouding the diagnosis of MCI, the focus of this study. This may have caused the sample to be less representative and created some spectrum bias, limiting the generalizability of the results. Finally, the study compared the Q*mci*-D only with the SMMSE-D. This was because the SMMSE is the most widely used short cognitive screen [34] and in the initial validation of the English language version of the Q*mci* the comparator was the SMMSE, allowing direct comparison with the results of that study [15]. The authors acknowledge the importance of future validation against other short, albeit longer, screens including the Dutch version of the Montreal cognitive assessment (MoCA) [35, 36], the Addenbrooke’s Cognitive Examination-Revised [37] and shorter instruments like the Mini-Addenbrooke’s Cognitive Examination (M-ACE) [38]*.* The authors also caution that screening for cognitive impairment continues to have challenges and in clinical practice [39] it remains only one part of a comprehensive assessment of cognition, and should not be relied upon exclusively.

## Conclusion

In conclusion, this study shows the concurrent validity of the Q*mci*-D against the SMMSE-D. The data suggests that the Q*mci*-D, although statistically significantly more accurate than the SMMSE-D, is limited in its ability to differentiate MCI from dementia. The results also suggest that the accuracy of both instruments at distinguishing MCI from NC was good although the Q*mci* was more accurate than the SMMSE in separating dementia from NC in a Dutch speaking population. Given this, albeit limited analysis in a small sample, as well as its brevity and ease of administration [15–17, 40], the Dutch version of the Q*mci*, the Q*mci*-D, appears useful as a short cognitive screen. Further research is now required to confirm these findings with a larger sample including other dementia subtypes and to compare the test to other cognitive screens including the MoCA and M-ACE.

## Abbreviations

ABCS 135: AB Cognitive Screen
ADAS-cog: Alzheimer’s Disease Assessment Scale – cognition
ANCOVA: Analysis of covariance
ANOVA: Analysis of variance
AUC: Area under the curve
DSM-IV: Diagnostic and Statistical Manual of Mental Disorders-IV
IQR: Inter-quartile range
M-ACE: Mini-Addenbrook’s Cognitive Examination
MCI: Mild cognitive impairment
MMSE: Mini-Mental State Examination
MoCA: Montreal cognitive assessment
NC: Normal cognition
NINCDS-ADRDA: National Institute of Neurological and Communicative Disorders and Stroke and the Alzheimer’s Disease and Related Disorders Association
SMMSE-D: Dutch translation of the Standardised Mini-Mental State Examination
Q*mci*: Quick mild cognitive impairment screen
ROC: Receiver operating characteristic
SD: Standard deviation
